# Phosphate of Lime

**Published:** 1880-08

**Authors:** 


### PHOSPHATE OF LIME.

On the *action of the phosphate of lime* Dr. Caspari remarks that it has a peculiar effect in improving the condition of the capillary circulation; that this effect is not due to the phosphoric acid contained in it; that preparations of iron, given in connection with the phosphate of lime, produce effects not obtainable by those preparations given alone; and that combinations of phosphorus and iron will not produce effects which may be derived from iron and phosphate of lime given together. The manner of operation of phosphate of lime he leaves for physiological chemistry to elucidate. He considers the phosphate of lime specially indicated in conditions characterized by hemorrhages due to the breaking down of capillary vessels. In support of this view he adduces cases of marked improvement under this treatment. In a case of acute parenchymatous nephritis, with more than the usual amount of hæmaturia, great benefit followed the daily administration of two drachms of the phosphate of lime. The hæmaturia was controlled, the blood finally disappearing from the urine altogether, and the nephritis rapidly progressing toward recovery. The author believes that the amelioration of the patient's condition was due to the influence of the phosphate of lime in improving the tone of the renal capillaries. A case of cystitis, with decomposing urine and cystorrhagia, was likewise favorably affected by the treatment. A case of menorrhagia was cured in the same way. The author has seen the best effects in chlorosis from a combination of iron preparations with the phosphate of lime; he has also seen the blood-streaked expectoration of catarrhal phthisis with cavities diminish conspicuously under a continued administration of the phosphate of lime.

